# Development of Highly-Active Catalysts toward Oxygen Reduction by Controlling the Shape and Composition of Pt–Ni Nanocrystals

**DOI:** 10.1021/acsami.3c10514

**Published:** 2023-10-13

**Authors:** Minghao Xie, Min Shen, Ruhui Chen, Younan Xia

**Affiliations:** † School of Chemistry and Biochemistry, Georgia Institute of Technology, Atlanta, Georgia 30332, United States; ‡ The Wallace H. Coulter Department of Biomedical Engineering, Georgia Institute of Technology and Emory University, Atlanta, Georgia 30332, United States

**Keywords:** alloy nanocrystals, shape control, composition control, oxygen reduction, fuel cell

## Abstract

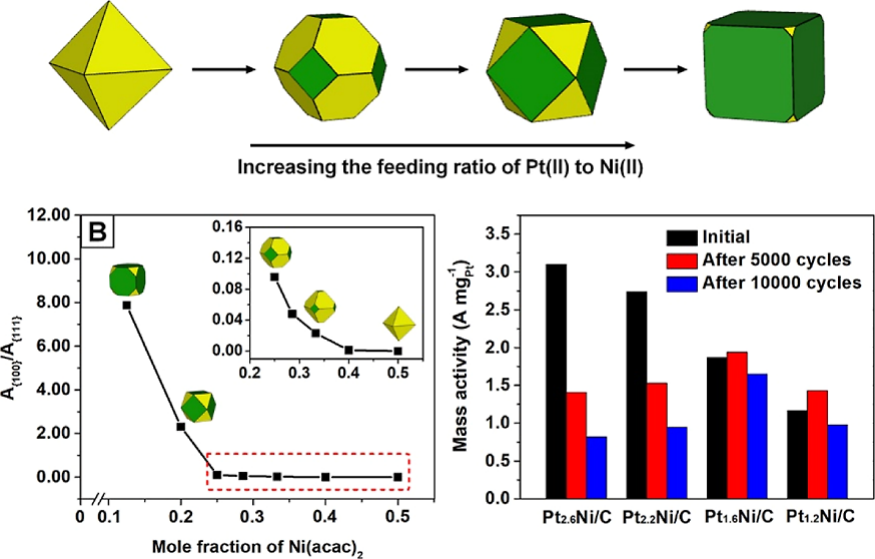

Electrocatalysts comprised of Pt–Ni alloy nanocrystals have garnered substantial attention due to their outstanding performance in catalyzing the oxygen reduction reaction (ORR). Herein, we present the synthesis of Pt–Ni nanocrystals with a variety of controlled shapes and compositions in an effort to investigate the impact of the Ni content on the formation of {111} facets and thereby the ORR activity. By completely excluding O_2_ from the reaction system, we could prevent the generation of Ni(OH)_2_ on the surface of the nanocrystals and thereby achieve a linear relationship between the atomic ratio of Pt to Ni in the nanocrystals and the feeding ratio of the precursors. The atomic ratio of Pt to Ni in the Pt–Ni nanocrystals was tunable within the range of 1.2–7.2, while their average sizes were kept around 9 nm in terms of edge length. In addition, a quantitative correlation between the area ratio of {111} to {100} facets and the feeding ratio of Pt(II) to Ni(II) was obtained by adjusting the mole fraction of the Ni(II) precursor in the reaction mixture. For the catalysts comprising octahedral nanocrystals, their specific ORR activities exhibited a positive correlation with the Pt/Ni atomic ratio. After the accelerated durability test, both specific and mass activity displayed a volcano-type trend with a peak value at a Pt/Ni atomic ratio of 1.6.

## Introduction

Polymer electrolyte membrane fuel cells (PEMFCs) offer a viable clean energy source for a range of applications, including decentralized power generation, transportation vehicles, and portable electronic devices.^1−3^ Despite being a well-established technology, widespread commercialization of PEMFCs is hindered by their ever-increasing cost.^2^ A large portion of the cost comes from the Pt-based electrocatalyst deposited on the cathode to alleviate the slow kinetics associated with the oxygen reduction reaction (ORR). At the moment, Pt is still the best candidate for the development of ORR electrocatalysts.^4^ To reduce the Pt content in PEMFC, extensive endeavors have been undertaken to substantially boost the catalytic activity of Pt-based nanoparticles through shape engineering (to present the most active facets on the surface) and composition variation.^5−11^ In the case of a structure-sensitive reaction like ORR, the specific activities (per unit surface area) of Pt in aqueous HClO_4_ solutions are identified to follow the trend of Pt(110) ≈ Pt(111) ≫ Pt(100).^12^ Consequently, it is anticipated that Pt nanocrystals with rhombic dodecahedral or octahedral shapes exhibit a much higher specific activity toward the ORR relative to that of their cubic counterparts. Alternatively, a substantial enhancement in specific activity can be achieved through alloying Pt with 3d transition metals like Fe, Co, or Ni.^6−11,13−16^ Notably, Stamenkovic and co-workers demonstrated that Pt–Ni alloy electrocatalysts exhibited significantly enhanced specific activities due to favorable changes to the electronic structure of Pt and thereby the adsorption energies of oxygen species.^13^ Specifically, the formation of Pt–Ni alloys leads to changes in the specific activities of the low-index facets, resulting in a new order of (111) ≫ (110) > (100).^10^ Motivated by this work, extensive research has been directed to the synthesis of Pt–Ni octahedral nanocrystals enclosed by {111} facets as the next-generation electrocatalyst toward ORR.^8,9,17,18^

The ORR activities of Pt-based bimetallic catalysts have a strong correlation with both the elemental composition and particle size.^6,7,15,19,20^ For instance, the Pt–Co nanocubes exhibited an enhancement in specific ORR activity with increasing Co content up to 25%, followed by a decrease with further enrichment of Co content.^19^ Experimental studies and density functional theory calculations have indicated that the highest ORR activity would be achieved at a Pt/Co atomic ratio of 3.^19^ On the other hand, the ORR activity of particle-based catalysts is highly dependent on their size.^21−23^ Previous research has verified that the specific activities of Pt and Pt-based alloy nanoparticles tend to be augmented with increasing particle size.^22,23^ However, the electrochemical surface area (ECSA) of the catalyst follows an opposite trend, and it decreases with increasing particle size. Therefore, the mass activity, which is determined by the balance between specific activity and the ECSA, reaches its maximum at an optimal particle size. In a previous study, we conducted the synthesis of Pt–Ni octahedral nanocrystals with controlled sizes and compositions and evaluated their ORR activities.^11^ It was discovered that the mass activities of these Pt–Ni octahedral nanocrystals reached a maximum when their edge length was controlled at around 9 nm. For the 9 nm Pt–Ni octahedral nanocrystals, both specific and mass activities exhibited a volcano-type plot, and the peak values were obtained at a Pt/Ni atomic ratio of 2.4.

Despite the awareness of optimal size, composition, and surface structure (i.e., type of facet) in maximizing the ORR activity of Pt–Ni nanocrystals, it was not easy to accomplish the target nanocrystals experimentally. Because of the technical difficulties in establishing an oxygen-free condition to exclude the generation of Ni(OH)_2_ on the surface of Pt–Ni nanocrystals throughout the synthesis, the atomic ratio of Pt to Ni in the nanocrystals did not follow a linear relationship with the feeding ratio between the Pt(II) and Ni(II) precursors.^9,11,24^ As such, it was difficult to quantify the effect of the Ni(II) amount on the generation of {111} facets and the shape of the Pt–Ni nanocrystals. Herein, we present a quantitative investigation of the syntheses and ORR activities of a series of Pt–Ni nanocrystals with a variety of controlled shapes and compositions. An airtight reflux setup was designed to realize O_2_-free synthesis and thus Ni(OH)_2_-free nanocrystal samples to elucidate the correlation between the feeding ratio of Pt(II) to Ni(II) and the area ratio of {111} to {100} facets of the Pt–Ni nanocrystals. We further evaluated the ORR activities of the Pt–Ni octahedral nanocrystals with an edge length around 9 nm, while their Pt/Ni atomic ratios were increased from 1.2 to 1.6, 2.2, and 2.6. For these octahedral nanocrystals, their specific and mass activities displayed a volcano-type plot after the durability test, with the peak corresponding to a Pt/Ni atomic ratio of 1.6.

## Experimental Section

### Chemicals and Materials

All chemicals were used as obtained from Sigma-Aldrich unless specified. The chemicals include oleylamine (OAm, 70%), oleic acid (OAc, 90%), benzyl ether (BE, ≥99.5%), platinum(II) acetylacetonate [Pt(acac)_2_, 97%], nickel(II) acetylacetonate [Ni(acac)_2_, ≥99.0%], tungsten hexacarbonyl [W(CO)_6_, 99.99%], and carbon black [Ketjen Black (KB) EC300JC]. Deionized water with a resistivity of 18.2 MΩ cm at room temperature was used for the preparation of all of the aqueous solutions.

### Synthesis of 9 nm Pt–Ni Polyhedral Nanocrystals in an Airtight Reflux Setup

**Figure 1:**
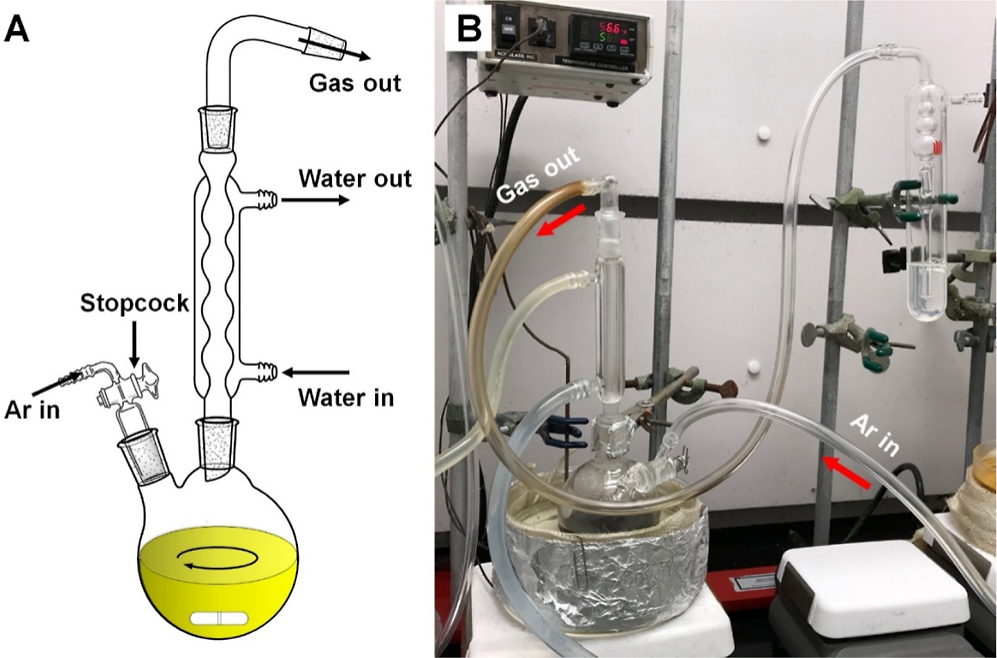
(A) Schematic illustration and (B) photograph showing the airtight reflux setup used for the preparation of Pt–Ni polyhedral nanocrystals without the production of Ni(OH)_2_ on the surface. The setup was designed for the removal and exclusion of O_2_ from the reaction system throughout the synthesis, with an oil tube attached to the gas outlet for the passage of Ar, the removal of O_2_, and the balance of pressure.

The setup was assembled from general laboratory apparatus, including a 50 mL 2-neck flask, a reflux condenser, and an oil tube, as shown in Figure 1. A horn tube with a straight stopcock plug was connected to one neck of the flask to provide an inlet for the flow of Ar, and the reflux condenser was connected to the oil tube to serve as an outlet for the flow of Ar, the removal of O_2_ from the setup, and the escape of CO during the synthesis. In a standard synthesis, Pt(acac)_2_ (0.051 mmol), Ni(acac)_2_ (0.051, 0.078, 0.102, 0.128, 0.153, 0.204, and 0.357 mmol for *x* = 1.2, 1.6, 2.2, 2.6, 3.2, 4.3, and 7.2, respectively), W(CO)_6_ (0.142 mmol), OAm (2 mL), OAc (1 mL), and 7 mL of BE were mixed in the 50 mL flask and heated to 60 °C under magnetic stirring with Ar protection to facilitate the dissolution of chemicals and the exclusion of O_2_. After 40 min, Ar purging was halted, and the flask was transferred into an oil bath and heated to 230 °C for 50 min. The reaction mixture was then immersed in an ice bath to rapidly lower its temperature. The Pt–Ni polyhedral nanocrystals were crushed out by adding toluene (10 mL) and ethanol (10 mL) sequentially. The supernatant was disposed of after centrifugation at 6000 rpm for 10 min. The as-obtained Pt–Ni polyhedral nanocrystals could be readily dispersed in toluene.

### Preparation of Pt–Ni/C

A suspension of the Pt–Ni nanocrystals in toluene was added dropwise into a suspension of KB carbon (with a Pt loading of *ca*. 20%) in toluene under ultrasonication for 3 h. The resulting Pt–Ni octahedra/C powder was collected by centrifugation at 12,000 rpm for 15 min, washed twice with toluene and ethanol, respectively, to help remove surface ligands and chemical species such as OAm, OAc, and WO_3_, and dryied for 30 min in an oven at 70 °C prior to electrochemical measurements.

### Characterizations

Transmission electron microscopy (TEM) images were acquired on an HT7700 microscope (Hitachi, Japan) operated at 120 kV. Inductively coupled plasma–mass spectrometry (ICP–MS, PerkinElmer, NexION 300Q) was used to quantify the metal contents in the catalysts. A PANalytical X’Pert PRO Alpha-1 diffractometer was employed to obtain X-ray diffraction (XRD) patterns using a 1.8 kW ceramic copper tube source. A KAlpha spectrometer (Thermo Scientific) was used for X-ray photoelectron spectroscopy (XPS) measurements with Al Kα X-ray (1486.6 eV) as the source.

### Electrochemical Measurements

Approximately 2 mg of the Pt–Ni octahedra/C catalyst was suspended in a mixture of water (2 mL), isopropyl alcohol (2 mL), and 16 μL of 5 wt % Nafion (Aldrich) via ultrasonication for 40 min. A 10 μL aliquot of this suspension was deposited and dried on a precleaned glassy carbon electrode (Pine Instruments). For reference and counter electrodes, a reversible hydrogen electrode (RHE) and a Pt mesh were employed, respectively. All potentials were measured relative to the RHE.

The ECSA of the catalyst was determined through the adsorption and desorption of hydrogen between 0.05 and 0.4 V, assuming 210 μC/cm^2^ for a hydrogen monolayer adsorbed on the Pt surface. Cyclic voltammetry (CV) measurements were performed at room temperature in 0.1 M HClO_4_ under an Ar flow with a sweeping rate of 50 mV s^–1^. Polarization curves were collected using a rotating disk electrode (RDE) in 0.1 M HClO_4_ under an O_2_ flow at room temperature with a sweeping rate of 10 mV s^–1^. The rotation speed of RDE was set at 1600 rpm. Before each measurement, several potential sweeps between 0.05 and 1.1 V were applied to the electrode until it reached a stable state. The kinetic current density was derived from the Koutecky–Levich equation
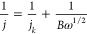
1where *j*, *B*, and ω represent the measured current density, a constant, and the rotation speed, respectively. A CHI600E potentiostat (CH Instrument, Austin, TX) was used to perform all of the electrochemical measurements. For the accelerated durability test (ADT), CV and polarization curves were collected after subjecting the catalyst to 5000 and 10,000 cycles of potential sweeping in the range of 0.6–1.1 V. These measurements were performed in a 0.1 M HClO_4_ solution saturated with Ar gas at room temperature with a scan rate of 100 mV s^–1^.

## Results and Discussion

### Synthesis and Characterization of Pt–Ni Nanocrystals

For the batch synthesis reported in the literature, Ni(OH)_2_ was always formed on the surface of Pt–Ni octahedral nanocrystals because no effort was made to completely exclude O_2_ from the reaction system.^9,24^ When the cooling process was initiated to quench the reaction, the decrease in gas pressure would draw external O_2_ into the reactor while the temperature was still high enough to induce the formation of Ni(OH)_2_ on the surface of the nanocrystals. The Ni(OH)_2_ could be removed through postsynthesis treatment with acetic acid (HAc), leading to observed increase in Pt/Ni atomic ratio for the alloy nanocrystals. In the present work, we addressed this issue by assembling an airtight reflux setup capable of eliminating the O_2_ from the reaction system at a temperature of 60 °C to dissolve all of the chemicals while keeping the precursors intact. Specifically, Ar was purged through the reaction system before all the chemicals were completely dissolved. The O_2_ dissolved in the solution should be blown away by the Ar gas. As a result, an inert atmosphere was created above the reaction solution before the reaction was initiated at a much higher temperature, 230 °C.

Figure 1A,B show a schematic and a photograph of the physical setup, respectively. Once the temperature was elevated to 230 °C, Ar purging was stopped by switching off the stopcock. Due to the decomposition of W(CO)_6_, CO was quickly released to act as a reducing agent for the creation of a reductive atmosphere above the reaction solution. The reflux condenser was maintained at a low temperature above the reactor, condensing the evaporated compounds back into the solution. The rapid color transition of the solution from yellow to dark brown suggests the formation of nanocrystals. After 50 min, the solution was cooled to room temperature, during which time the pressure in the reactor gradually dropped. The oil tube at the end of the gas flow served as a pressure balance, preventing the backflow of air into the reactor and thus maintaining a reductive atmosphere during the cooling process. Under this set of experimental conditions, we could obtain 9.3 nm Pt–Ni octahedral nanocrystals at a throughput of 10 mg per batch without the generation of Ni(OH)_2_ on the surface of the nanocrystals (Figure S1).

**Figure 2:**
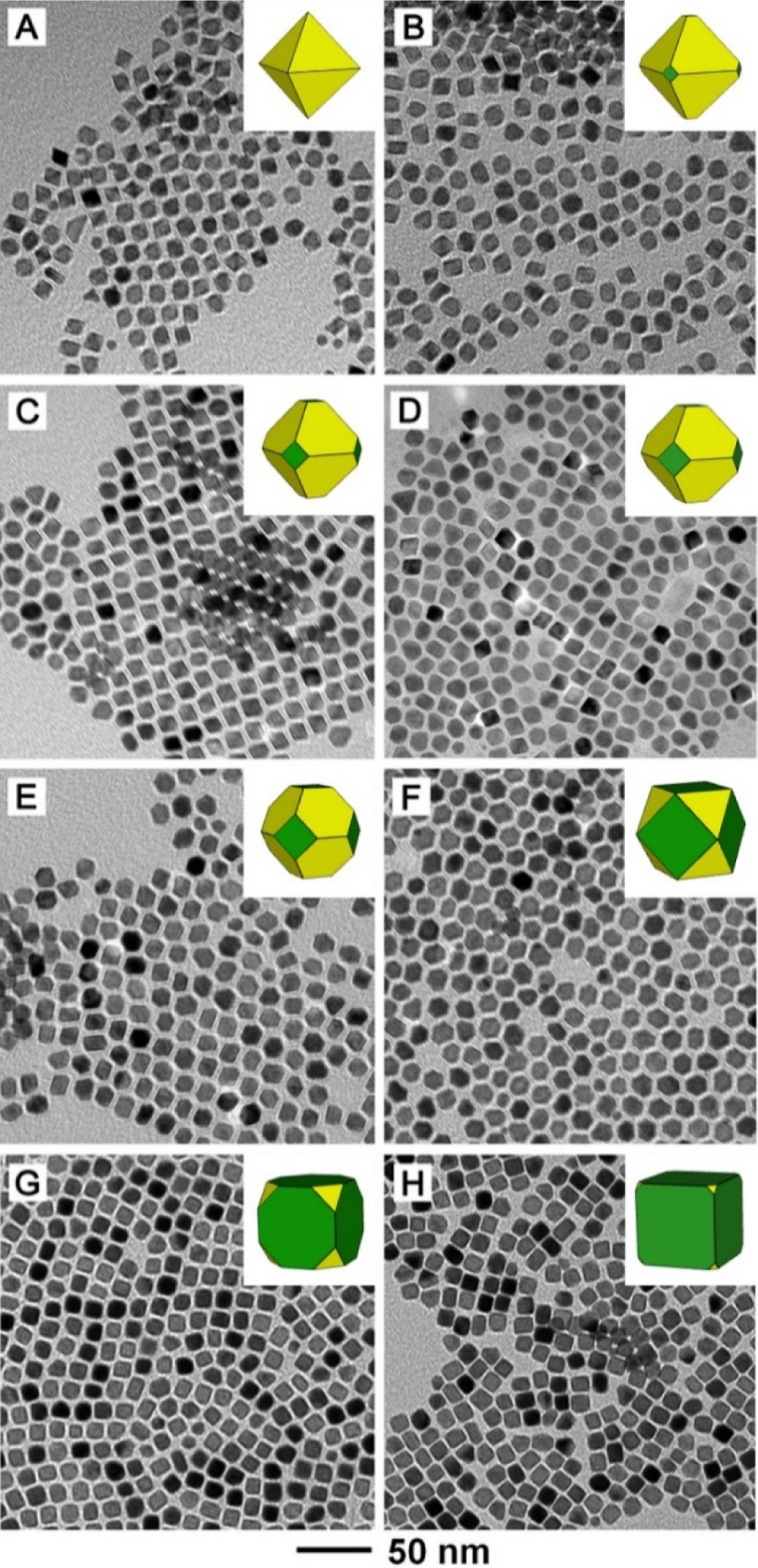
TEM images of Pt–Ni polyhedral nanocrystals synthesized using the standard protocol except for the addition of different amounts of Ni(acac)_2_ to give Pt(II) to Ni(II) feeding ratios of (A) 1, (B) 1.5, (C) 2, (D) 2.5, (E) 3, (F) 4, and (G) 7. The sample in (H) was prepared in the absence of Ni(acac)_2_. The insets in (A–H) show models of the corresponding polyhedral nanocrystals. As the feeding ratio of Pt(II) to Ni(II) was increased, the resultant Pt–Ni nanocrystals changed from (A) octahedra to (B–E) truncated octahedra, (F) cuboctahedra, (G) truncated cubes, and (H) cubes, suggesting the role of Ni in inducing the formation of {111} facets.

In the generation of Pt–Ni octahedral nanocrystals, the incorporation of Ni atoms varied the binding preference of CO from{100} to {111} facets, thereby favoring the production of an octahedral shape.^8,9^ The aforementioned reactor enabled us to quantitatively study the correlations between the Pt/Ni atomic ratio in the resultant nanocrystals and their shapes by adding different amounts of the Ni(II) precursor into the reaction solution with all other parameters unchanged. Figure 2 shows TEM images of the as-obtained Pt/Ni polyhedral nanocrystals. The feeding ratio of Pt(II) to Ni(II) was increased from 1 to 1.5, 2, 2.5, 3, 4, and 7. The shapes of the Pt–Ni nanocrystals were derived from the projections of the nanocrystals under TEM. Figure S2 shows TEM images and the corresponding polyhedral models of individual nanocrystals to better differentiate the {111} and {100} facets. Octahedral nanocrystals with sharp corners were obtained only when the mole fraction of Ni(acac)_2_ in the precursors reached 0.5 (Figure 2A). When this ratio decreased to 0.4, the corners of the octahedral nanocrystals became slightly truncated, exposing {100} facets on the surface (Figure 2B). The truncated octahedral shape could still be preserved as long as the mole fraction of Ni(acac)_2_ in the precursor mixture was higher than 0.2 (Figure 2C–E). If the mole fraction of Ni(acac)_2_ was reduced to 0.2, then Pt–Ni nanocrystals with a cuboctahedral shape were obtained (Figure 2F). Further decreasing the mole fraction of Ni(II) resulted in the production of Pt–Ni cubic nanocrystals with truncated corners (Figure 2G). In the absence of Ni(acac)_2_, the resultant Pt nanocrystals took on cubic shape, as shown in Figure 2H.

### Mechanistic Understanding of the Synthesis

**Figure 3:**
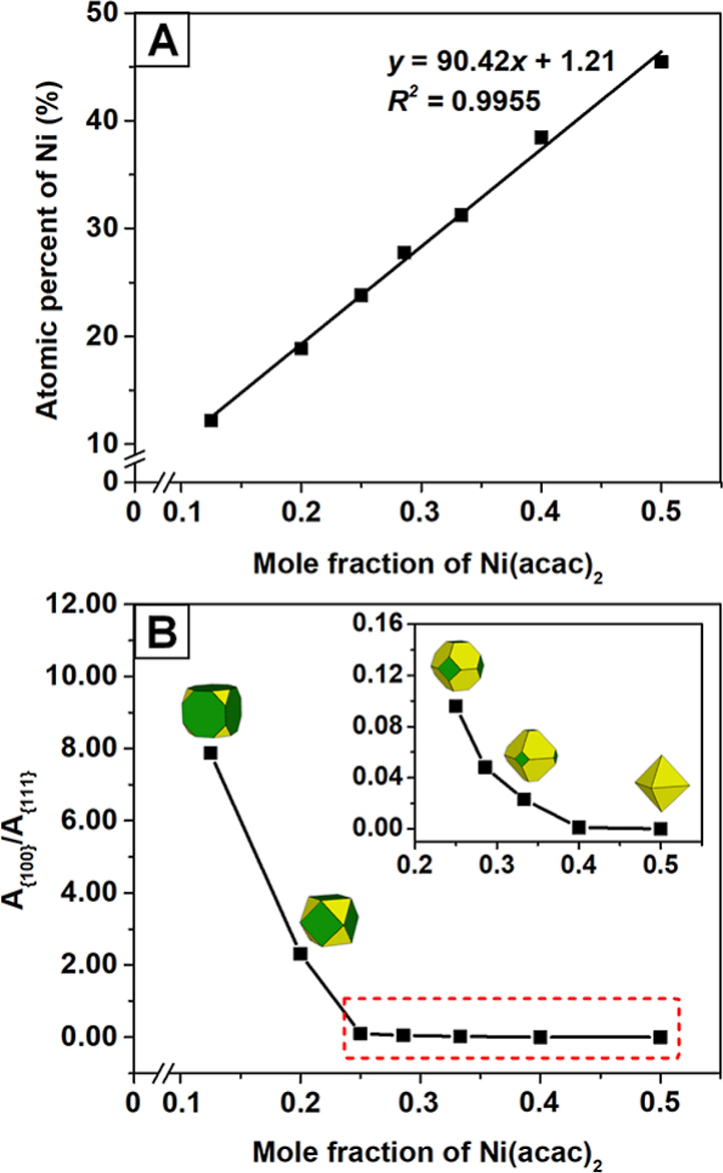
Plots of (A) atomic percent of Ni in the Pt–Ni polyhedral nanocrystals and (B) ratio between the areas of {100} and {111} facets as a function of the mole fraction of Ni(acac)_2_ in the precursor mixture.

The correlation between the atomic percent of Ni in the nanocrystals (as measured using ICP–MS) and the mole fraction of Ni(acac)_2_ in the precursor mixture is shown in Figure 3A. Different from all of the syntheses of Pt–Ni octahedral nanocrystals reported in the literature, a linear relationship was achieved due to the exclusion of Ni(OH)_2_ formation. To quantify the effect of Ni on inducing the formation of {111} facets, we use the ratio between the facet areas of {100} and {111} (*A*_{100}_/*A*_{111}_) to describe the shape of Pt–Ni polyhedral nanocrystals as a function of the mole fraction of Ni(acac)_2_ in the precursor mixture. The definition and calculation of *A*_{100}_/*A*_{111}_ for three types of nanocrystals with truncated octahedral, cuboctahedral, and truncated cubic shapes are illustrated in Figure S3–S5. As a reference, the *A*_{100}_/*A*_{111}_ for a cuboctahedron was calculated to be 2.309, above and below which the values indicate a truncated cube and a truncated octahedron, respectively (Table S1). As shown in Figure 3B, the *A*_{100}_/*A*_{111}_ of the as-obtained nanocrystals with different atomic percents of Ni were calculated to quantify the shape alteration. The result indicates that the incorporation of Ni can substantially decrease the value of *A*_{100}_/*A*_{111}_ even if a small amount of Ni is present in the nanocrystals. Specifically, increasing the mole fraction of Ni(acac)_2_ from 0.125 to 0.2 resulted in a 70.7% decrease in *A*_{100}_/*A*_{111}_, changing the shape from a truncated cube to a cuboctahedron. When the mole fraction was increased to 0.25, the value of *A*_{100}_/*A*_{111}_ dropped below 0.1, corresponding to the formation of a truncated octahedral shape. The variations of *A*_{100}_/*A*_{111}_ below 0.1 correspond to the octahedral shape with different degrees of truncation, suggesting that the truncation could be decreasingly diminished by increasing the mole fraction of Ni(acac)_2_ up to 0.4. Eventually, *A*_{100}_/*A*_{111}_ could be minimized to 0 at a mole fraction of 0.5 for Ni(acac)_2_. In practical applications, this quantitative relationship can be used to synthesize Pt–Ni nanocrystals with a precise Pt/Ni atomic ratio and a desired facet area ratio by estimating the required quantity of Ni(acac)_2_ for the synthesis.

**Figure 4:**
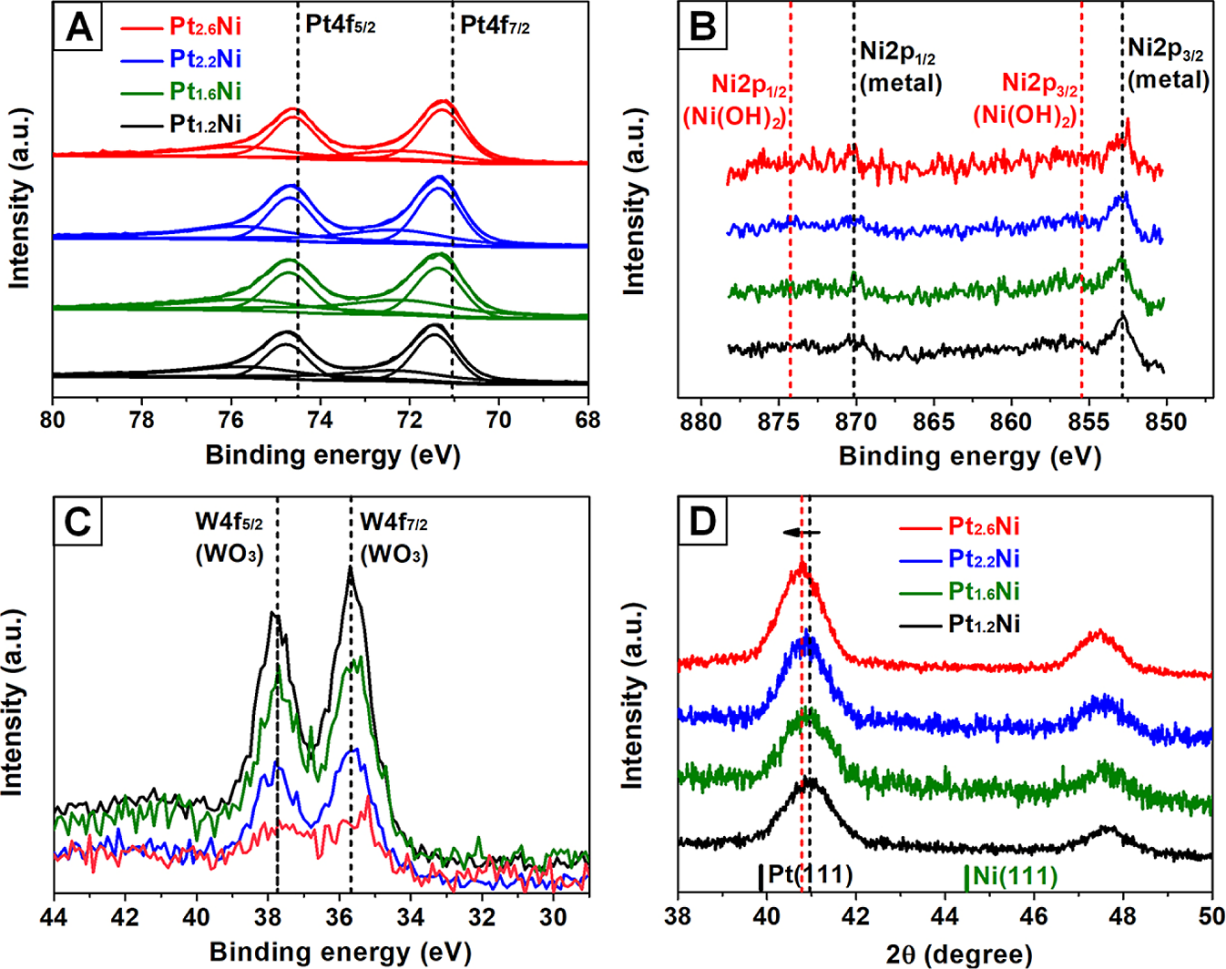
(A–C) XPS spectra of Pt_1.2_Ni, Pt_1.6_Ni, Pt_2.2_Ni, and Pt_2.6_Ni octahedral nanocrystals: (A) Pt 4f, (B) Ni 2p, and (C) W 4f. (D) XRD patterns of Pt_1.2_Ni, Pt_1.6_Ni, Pt_2.2_Ni, and Pt_2.6_Ni octahedral nanocrystals. The diffraction peaks were shifted gradually to higher 2θ values as the content of Ni in Pt_*x*_Ni was increased. Black bars: JCPDS #04-0802 (Pt); Green bars: JCPDS #01-1258 (Ni).

We used XPS to investigate the electronic structure and surface species of the Pt–Ni octahedral nanocrystals with different Pt/Ni atomic ratios. The Pt 4f spectrum (Figure 4A) signifies the prevalence of zerovalent Pt in all the as-obtained samples. When compared with pure Pt, the binding energy of the Pt 4f peaks in the Pt–Ni nanocrystals was upshifted by 0.52, 0.49, 0.47, and 0.42 eV for samples with Pt/Ni atomic ratios of 1.2, 1.6, 2.2, and 2.6, respectively.^25^ The result indicates that alloying Pt with another metal, such as Ni, could alter the electronic structure of Pt, and this effect was enhanced with an increased amount of Ni in the alloys.^26^ For the Ni 2p XPS spectra (Figure 4B), only zerovalent Ni metal was observed in all samples, confirming the absence of Ni(OH)_2_ on the surface even for nanocrystals with a high atomic percent of Ni. Meanwhile, the W 4f spectra (Figure 4C) reveal the existence of WO_3_ in the samples, suggesting that W decomposed from W(CO)_6_ might also act as a reducing agent for the reaction.^8^ According to previous reports, the WO_3_ could be easily removed from the surface by repeatedly washing with toluene or HAc, indicating that W was not integrated into the crystal lattice of the nanocrystals.^8,9,24^ The amount of WO_3_ on the surface increased with the atomic percent of Ni, indicating the possible role played by W in the reduction of Ni(II) under the experimental conditions used. Figures S6 and 4D give the XRD patterns of the Pt–Ni octahedral nanocrystals with different compositions, which resembled those of Pt with a face-centered cubic (fcc) structure (JCPDS #04-0802). The diffraction peaks were slightly shifted to higher 2θ values in comparison with those of fcc Pt, which can be ascribed to a decrease in lattice spacing for the Pt–Ni octahedral nanocrystals resulting from the substitution of Pt atoms (atomic radius: 1.39 Å) with smaller Ni atoms (atomic radius: 1.24 Å) in the crystal lattice. In addition, the diffraction peaks gradually shifted toward higher 2θ values as the Ni content in the Pt–Ni octahedral nanocrystals increased, which was in line with that observed in previous reports.^11^ It is worth noting that the positions of the diffraction peaks from different samples did not follow Vegard’s law exactly, and the discrepancy could be attributed to the possible formation of amorphous Ni nanoparticles in the reaction system and thus the deviation of composition from the corresponding Pt/Ni atomic ratio. However, we did not observe such amorphous Ni nanoparticles in all of the as-obtained samples under TEM. If such amorphous Ni nanoparticles were formed, they should take a spherical shape, strikingly different from the polyhedral shape exhibited by the Pt–Ni alloy nanocrystals. In addition, the XPS results clearly reveal the shift of Pt 4f peaks to higher binding energy with an increased atomic ratio of Ni/Pt, suggesting that Ni was largely incorporated into the lattice to form Pt–Ni alloys.

### Electrochemical Evaluation of the Pt–Ni Octahedral Nanocrystals

**Figure 5:**
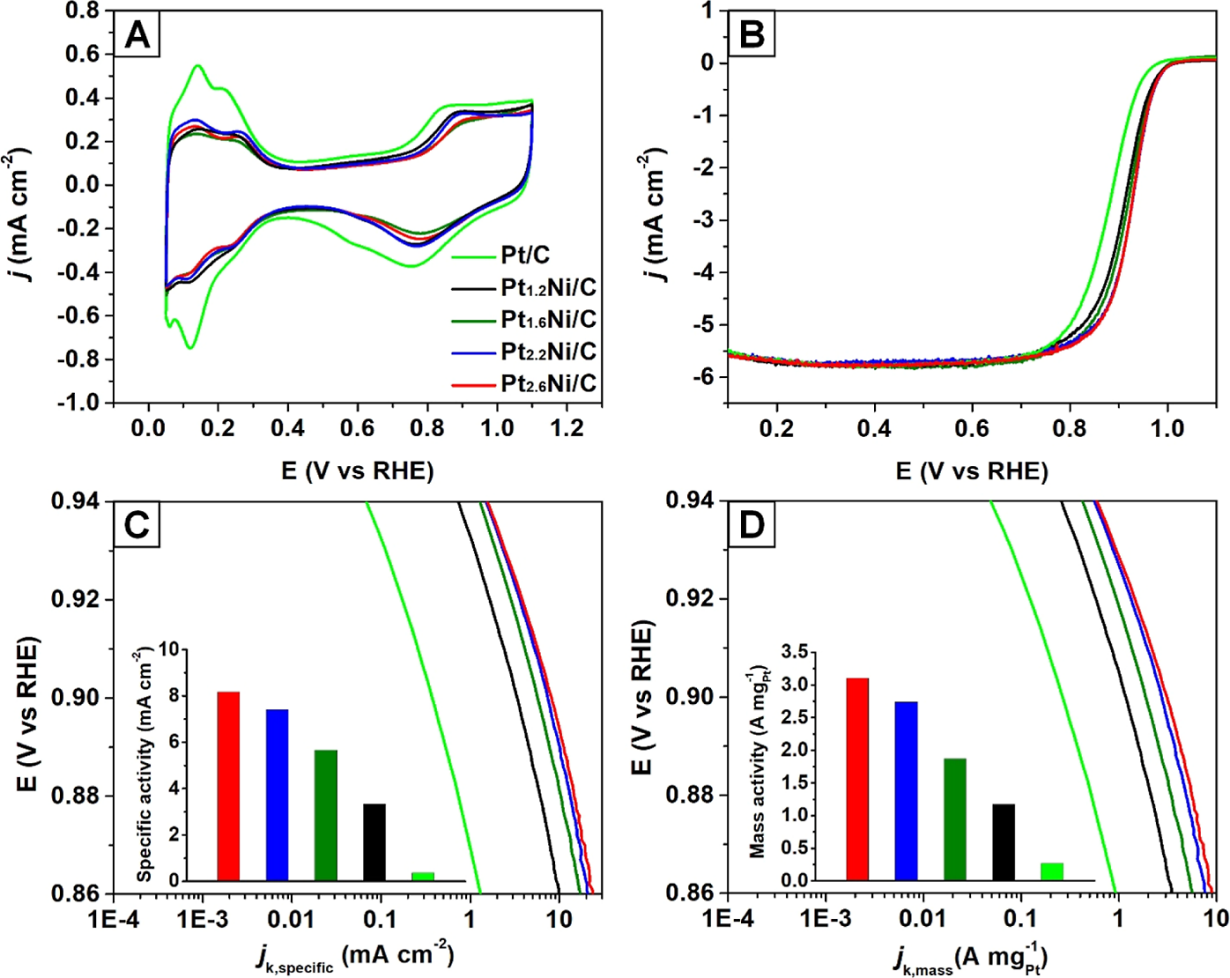
Comparison of the electrocatalytic properties of the Pt–Ni/C octahedral catalysts tested using the liquid half-cell. (A) CV curves of the catalysts recorded in Ar-saturated 0.1 M HClO_4_ solutions. Scanning rate: 50 mV s^–1^. (B) Positive-going ORR polarization curves of the catalysts recorded in O_2_-saturated 0.1 M HClO_4_ solutions. Scanning rate = 10 mV s^–1^. Rotation speed: 1600 rpm. The currents were normalized to the geometric area of the RDE (0.196 cm^2^). (C) Specific activity and (D) Pt mass activity at 0.9 V_RHE_ based on the ECSAs determined from the charges associated with *H*_UPD_ and Pt mass loading, respectively.

The Pt–Ni octahedral nanocrystals were deposited on carbon black (Pt–Ni/C, Figure S7) to benchmark their electrochemical properties against a commercial Pt/C (20 wt % 3.2 nm Pt particles supported on Vulcan XC72, Premetek Co.). Figure 5 shows the electrochemical properties of Pt–Ni/C catalysts with Pt/Ni atomic ratios of 1.2, 1.6, 2.2, and 2.6, respectively. Figure 5A shows the CV curves used to calculate the ECSAs of the catalysts according to the hydrogen adsorption/desorption (*H*_UPD_) features between 0.05 and 0.4 V_RHE_. The ECSAs were in the range 35–40 cm^2^ g_Pt_^–1^ (Table S2). The comparable ECSAs indicated that these Pt–Ni octahedral nanocrystals were indeed synthesized with a consistent size of approximately 9 nm in edge length.

Since the improved activities of the Pt alloys arise from the incorporation of transition metals, the Ni content in the Pt–Ni/C catalysts is expected to play a major role in enhancing their ORR activity.^11^Figure 5B exhibits the positive-going ORR polarization curves of the catalysts. The specific (*j*_k,specific_) and mass activities (*j*_k,mass_) were obtained from the kinetic current density (*j*_k_) normalized to ECSA and Pt mass, respectively. The kinetic current density was derived, in turn, from the Koutecky–Levich equation. Compared with the commercial Pt/C catalyst, the specific activities at 0.9 V_RHE_ of Pt–Ni/C were all significantly improved (Figure 5C). This enhancement could be ascribed to the ligand and strain effects resulting from the integration of Ni atoms into the Pt lattice and the electronic interaction between the two metals, respectively.^8,9,11^ According to previous studies, the ORR kinetics on a bulk Pt–Ni{111} surface can be maximized when the atomic percent of Ni approaches 25%, delivering optimized oxygen adsorption free energy (*E*_0_) to realize a maximal specific activity.^10,11^ When the amount of Ni surpasses this optimal value, *E*_0_ will not be strong enough to facilitate the breaking of the O=O bond. As a result, Pt_2.6_Ni/C with an atomic percent of Ni of 27.8% exhibited the highest specific activity. When the atomic percent of Ni was increased from 27.8 to 31.3, 38.5, and 45.5%, the specific activity dropped by 9.2, 30.5, and 59.1%, respectively, revealing the gradually increased negative effect of Ni with atomic percent above the optimal value. Given that the ECSAs of Pt–Ni octahedral nanocrystals in these catalysts were in close proximity to each other, the Pt-based mass activity exhibited a similar trend as well (Figure 5D).

**Figure 6:**
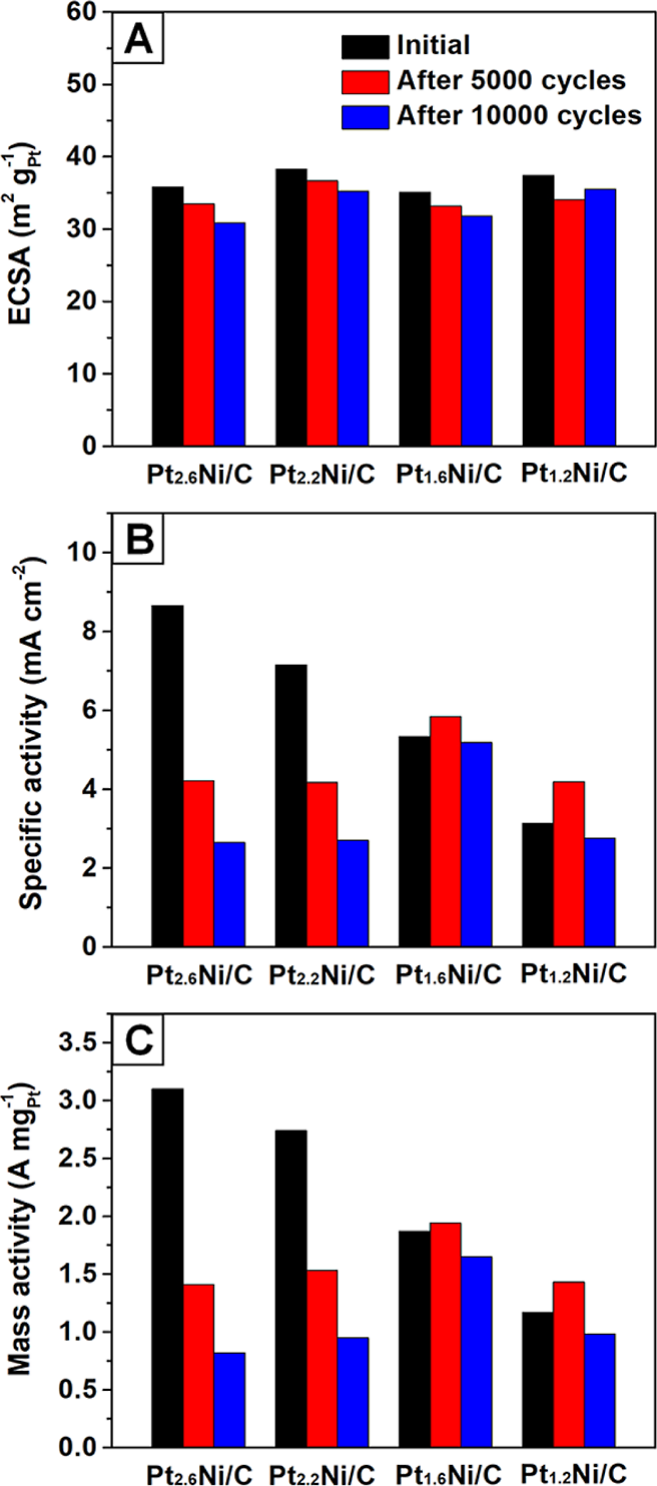
(A) Specific ECSA and (B) specific and (C) mass activity of the Pt–Ni/C octahedral catalysts at 0.9 V_RHE_ before and after different potential cycles in 0.1 M HClO_4_. Each set of durability tests was conducted using the same Pt–Ni/C electrode.

In addition to the assessment of catalytic activity, we performed ADT to investigate the long-term stability of the Pt–Ni/C catalysts (Figures S8 and S9). After 10,000 cycles of ADT, the ECSAs dropped by 5.1, 9.4, 8.1, and 13.7%, for samples with the Pt/Ni atomic ratios of 1.2, 1.6, 2.2, and 2.6, respectively (Figure 6A and Table S2). The reduced degradation of ECSA for samples with a higher atomic percent of Ni indicates enhanced hydrogen adsorption/desorption on the surface, which was likely due to the leaching of surface Ni for the exposure of Pt atoms. The specific activities of Pt_1.2_Ni/C, Pt_1.6_Ni/C, Pt_2.2_Ni/C, and Pt_2.6_Ni/C were reduced by 11.8, 2.6, 62.2, and 69.4%, respectively, after 10,000 cycles (Figure 6B). The severe loss of specific activity for Pt_2.2_Ni/C and Pt_2.6_Ni/C could be ascribed to the substantial deviation of the atomic percent of Ni from the optimal value under the highly corrosive environment. Figure S10 shows a TEM image of Pt_2.6_Ni/C after ADT, indicating observable shape alteration and minor agglomeration of the nanocrystals. These changes contributed to severe degradation in specific activity and a drop in ECSA during ADT. Notably, the specific activity after ADT resulted in a volcano-type plot, in which the peak value corresponds to a Pt/Ni atomic ratio of 1.6 before the ADT. The mass activity exhibited the same volcano trend due to the similar ECSAs of the samples (Figure 6C). The result suggests that Pt_1.6_Ni/C after ADT featured an atomic percent of Ni (22.8%) close to the optimal value, which was verified by ICP–MS.

## Conclusions

We successfully synthesized Pt–Ni nanocrystals with controlled shapes and compositions while excluding the formation of Ni(OH)_2_ on the surface. With the use of an airtight reflux setup, the reaction system could completely eliminate O_2_ to ensure a reductive atmosphere throughout the synthesis, helping achieve a linear relationship between the feeding ratio of Pt(II) to Ni(II) and the resulting Pt/Ni atomic ratio in the nanocrystals. The shapes of the Pt–Ni nanocrystals could be tuned from octahedron to truncated octahedron, cuboctahedron, truncated cube, and cube by introducing different amounts of Ni(acac)_2_ into the reaction solution. More importantly, a correlation between the feeding ratio of Pt(II) to Ni(II) and the facet area ratio of the resultant Pt–Ni nanocrystals was obtained, shedding light on the effect of Ni incorporation on the formation of {111} facets. Electrochemical evaluations demonstrated that the specific ORR activities of Pt–Ni octahedral nanocrystals dropped by 9.2, 30.5, and 59.1% when the atomic percent of Ni was increased from 27.8 to 31.3, 38.5, and 45.5%, respectively, unraveling the increasingly unfavorable effect of Ni with atomic percent above the optimal value of 25%. The specific and mass activity after the ADT exhibited a volcano-type trend, with a peak value corresponding to a Pt/Ni atomic ratio of 1.6. The results from this study offer valuable insights for the future development and synthesis of advanced ORR catalysts utilizing Pt–Ni alloy nanocrystals.

## Supporting Information Available

TEM images, XRD, ICP–MS, fact area ratio calculations, and electrochemical performance of the catalysts (PDF)
