# Supplementary material for: Development of Highly-Active Catalysts toward Oxygen Reduction by Controlling the Shape and Composition of Pt–Ni Nanocrystals
Source: ACS Appl Mater Interfaces. 2023 Oct 13;15(42):49146–53. doi: 10.1021/acsami.3c10514 (PMC10614184; doi:10.1021/acsami.3c10514)
Supplement: Supplementary file 1 — am3c10514_si_001.pdf [file am3c10514_si_001.pdf]

Supporting Information

Development of Highly-Active Catalysts toward Oxygen Reduction by Controlling the Shape and Composition of Pt-Ni Nanocrystals

Minghao Xie,[a] *Min Shen,*[b] *Ruhui Chen,*[a] *and Younan Xia*[a,b*]

[a]School of Chemistry and Biochemistry, Georgia Institute of Technology, Atlanta, Georgia 30332, United States

[b]The Wallace H. Coulter Department of Biomedical Engineering, Georgia Institute of Technology and Emory University, Atlanta, Georgia 30332, United States

[*]Corresponding author. E-mail: younan.xia@bme.gatech.edu

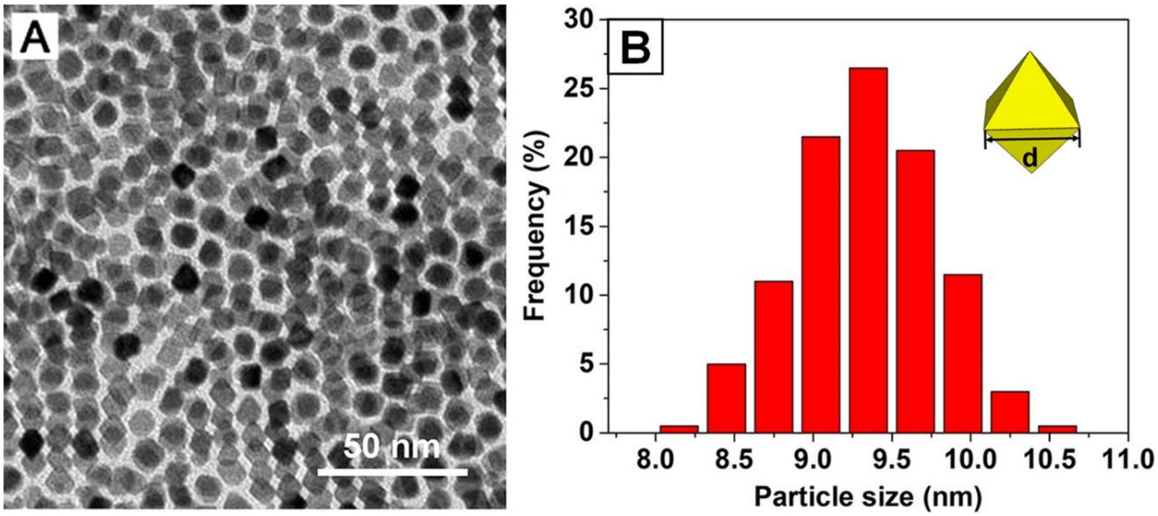

Figure S1. (A) TEM image and (B) size distribution of the $Pt_{2.6}Ni$ octahedral nanocrystals with an average edge length of 9.3 ± 0.5 nm, which were synthesized using the standard protocol. The inset in panel (B) shows a schematic of the octahedron and the definition of edge length (d).

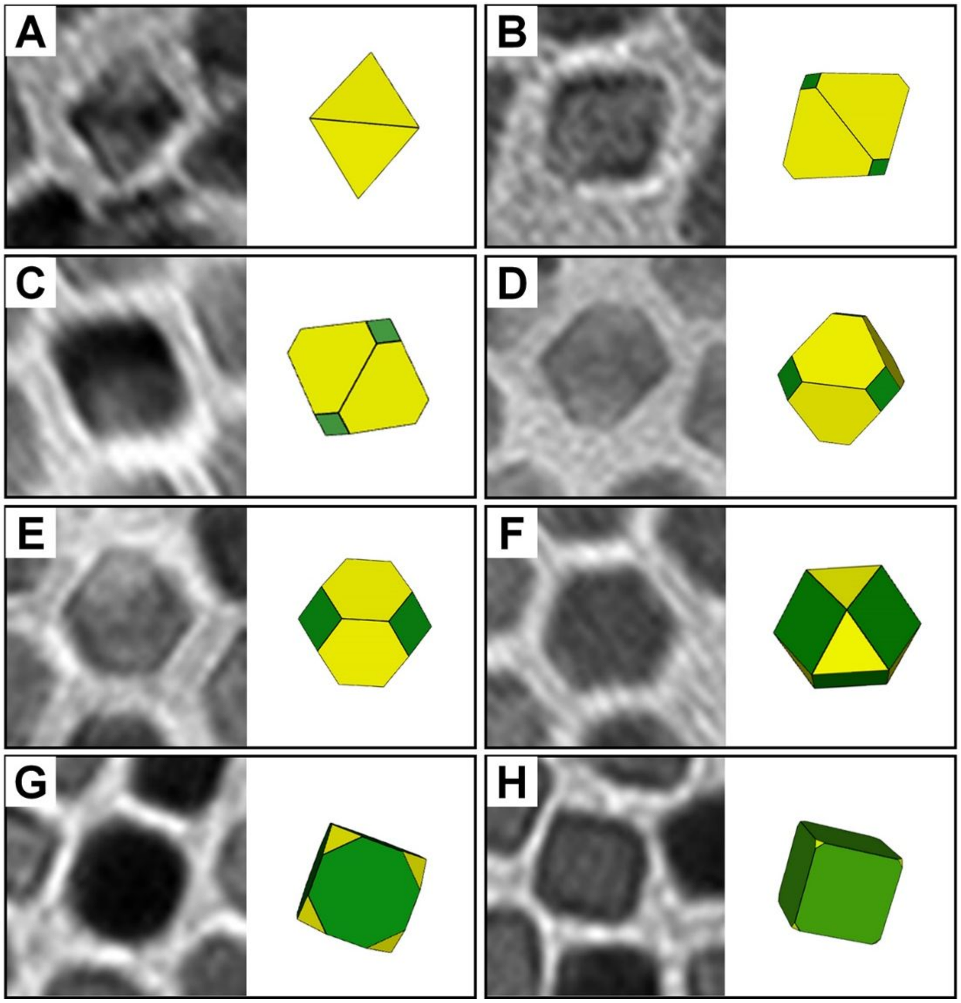

Figure S2. Magnified TEM images of Pt-Ni polyhedral nanocrystals and the models of corresponding polyhedral nanocrystals prepared using the standard protocol except for the use of different amounts of Ni(acac)$_2$ to give Pt(II) to Ni(II) feeding ratios of (A) 1, (B) 1.5, (C) 2, (D) 2.5, (E) 3, (F) 4, and (G) 7, respectively. The sample in (H) was prepared in the absence of Ni(acac)$_2$.

Calculation of the facet area ratio for a truncated octahedron:

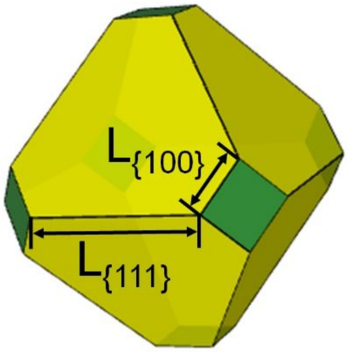

Figure S3. Schematic illustration showing the definitions of the edge length of {100} facet ($L_{\{100\}}$), and the edge length of {111} facets ($L_{\{111\}}$).

The area of {100} facet was obtained from Equation (1):

$$A_{\{100\}} = {L_{\{100\}}}^2 \tag{1}$$

where $L_{\{100\}}$ is the edge length of {100} facet. The area of {111} facet was obtained from Equation (2):

$$A_{\{111\}} = \frac{\sqrt{3}}{4}{L_{\{111\}}}^2 + \frac{\sqrt{3}}{4}{L_{\{100\}}}^2 + \sqrt{3}L_{\{100\}}L_{\{111\}} \tag{2}$$

where $L_{\{111\}}$ is the edge length of {111} facet. The facet area ratio between {100} and {111} facets was obtained from Equation (3):

$$\frac{A_{\{100\}}}{A_{\{111\}}} = \frac{{L_{\{100\}}}^2}{\frac{\sqrt{3}}{4}{L_{\{111\}}}^2 + \frac{\sqrt{3}}{4}{L_{\{100\}}}^2 + \sqrt{3}L_{\{100\}}L_{\{111\}}} = \frac{1}{\frac{\sqrt{3}}{4}\left(\frac{L_{\{111\}}}{L_{\{100\}}}\right)^2 + \frac{\sqrt{3}}{4} + \sqrt{3}\frac{L_{\{111\}}}{L_{\{100\}}}} = \frac{\sqrt{3}}{3{a_{\{111\}/\{100\}}}^2 + 3 + 12a_{\{111\}/\{100\}}} \tag{3}$$

where $a_{\{111\}/\{100\}}$ is the edge length ratio between {111} and {100} facets obtained from $a_{\{111\}/\{100\}} = \frac{L_{\{111\}}}{L_{\{100\}}}$

Calculation of the facet area ratio for a cuboctahedron:

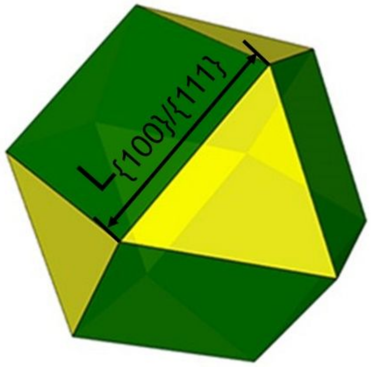

Figure S4. Schematic illustration showing the definitions of the edge length of {100} and {111} facets ($L_{\{100\}/\{111\}}$).

The area of {100} facet was obtained from Equation (4):

$$A_{\{100\}} = L_{\{100\}/\{111\}}{}^2 \tag{4}$$

where $L_{\{100\}/\{111\}}$ is the edge length of {100} facet. The area of {111} facet was obtained from Equation (5):

$$A_{\{111\}} = \frac{\sqrt{3}}{4} L_{\{100\}/\{111\}}{}^2 \tag{5}$$

where $L_{\{100\}/\{111\}}$ is the edge length of {111} facet. The facet area ratio between {100} and {111} facets was obtained from Equation (6):

$$\frac{A_{\{100\}}}{A_{\{111\}}} = \frac{L_{\{100\}/\{111\}}{}^2}{\frac{\sqrt{3}}{4} L_{\{100\}/\{111\}}{}^2} = 2.309 \tag{6}$$

Calculation of the facet area ratio for a truncated cube:

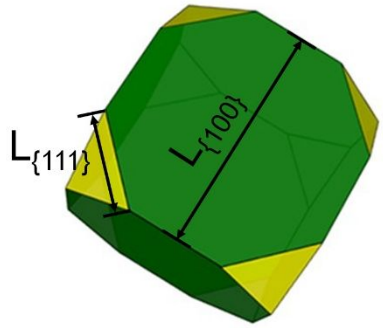

Figure S5. Schematic illustration showing the definitions of the edge length of {100} facet ($L_{\{100\}}$), and the edge length of {111} facets ($L_{\{111\}}$).

The area of {111} facet was obtained from Equation (7):

$$A_{\{111\}} = \frac{\sqrt{3}}{4} L_{\{111\}}{}^2 \tag{7}$$

where $L_{\{111\}}$ is the edge length of {111} facet. The area of {100} facet was obtained from Equation (8):

$$A_{\{100\}} = L_{\{100\}}{}^2 - L_{\{111\}}{}^2 \tag{8}$$

where $L_{\{100\}}$ is the edge length of {100} facet. The facet area ratio between {100} and {111} facets was obtained from Equation (9):

$$\frac{A_{\{100\}}}{A_{\{111\}}} = \frac{L_{\{100\}}{}^2 - L_{\{111\}}{}^2}{\frac{\sqrt{3}}{4} L_{\{111\}}{}^2} = \frac{4\sqrt{3}}{3}\left(a_{\{100\}/\{111\}}{}^2 - 1\right) \tag{9}$$

where $a_{\{100\}/\{111\}}$ is the edge length ratio between {100} and {111} facets obtained from $a_{\{100\}/\{111\}} = \frac{L_{\{100\}}}{L_{\{111\}}}$

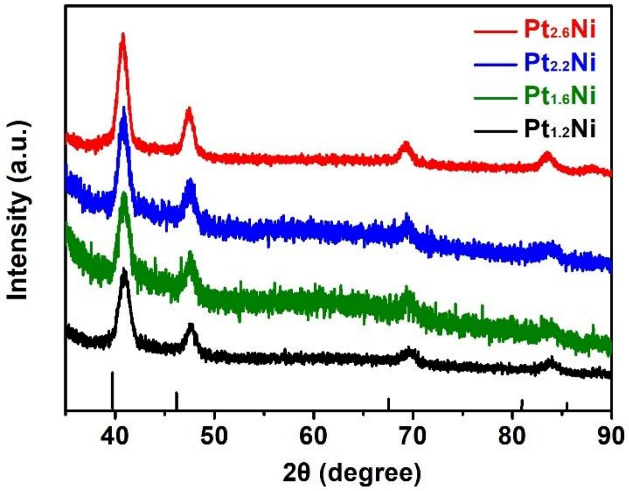

Figure S6. XRD patterns of the $Pt_{1.2}Ni$, $Pt_{1.6}Ni$, $Pt_{2.2}Ni$, and $Pt_{2.6}Ni$ octahedral nanocrystals. Black bars: JCPDS #04-0802 (Pt).

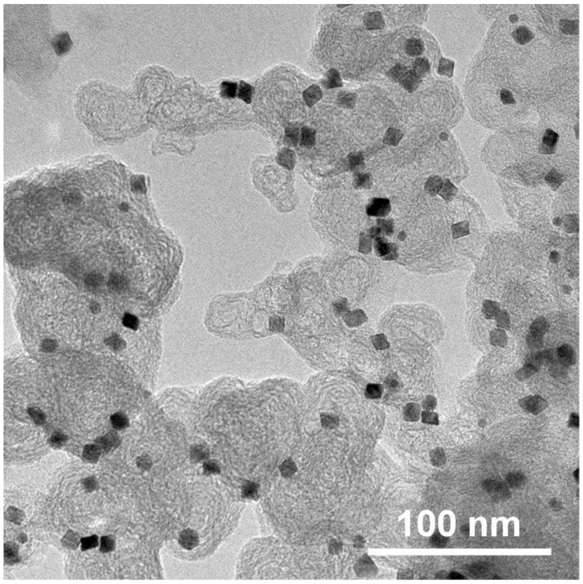

Figure S7. TEM image of the $Pt_{2.6}Ni$ octahedral nanocrystals supported on carbon black at a Pt loading of *ca.* 20 wt%.

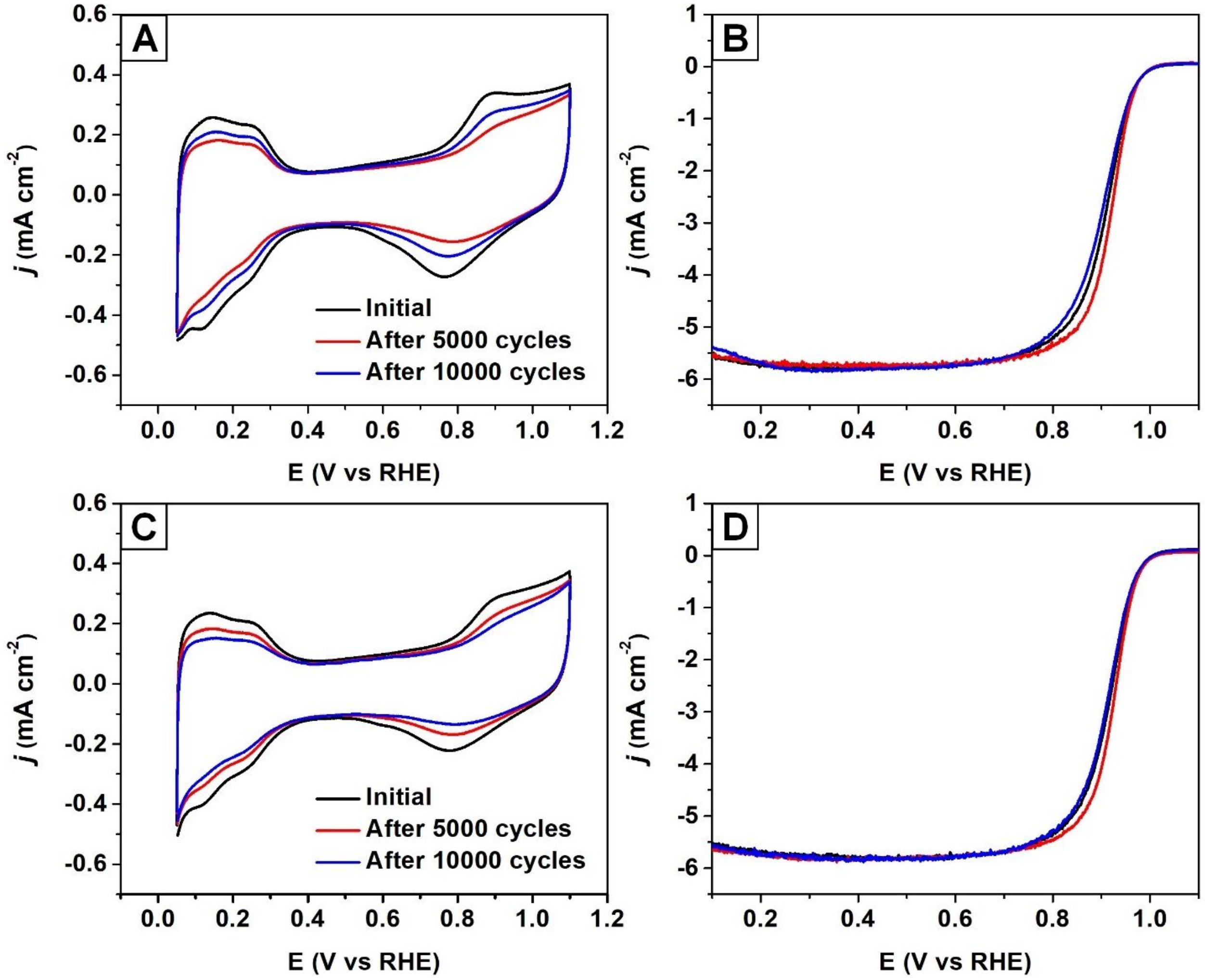

Figure S8. (A) CV and (B) ORR polarization curves recorded from the $Pt_{1.2}Ni/C$ before and after different cycles of ADT. (C) CV and (D) ORR polarization curves recorded from the $Pt_{1.6}Ni/C$ before and after different cycles of ADT. The currents were normalized to the geometric area of the rotating disk electrode.

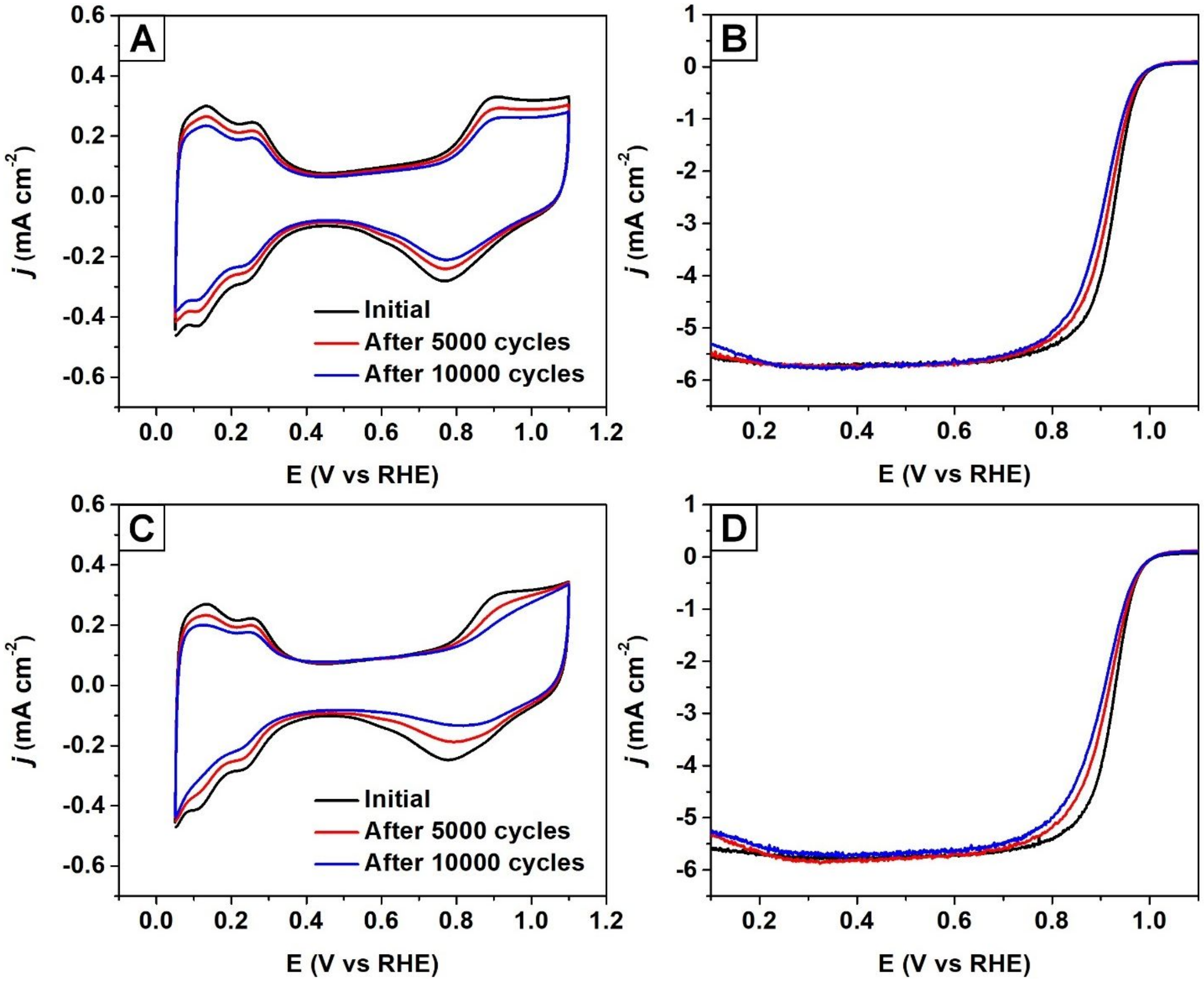

Figure S9. (A) CV and (B) ORR polarization curves recorded from the $Pt_{2.2}Ni/C$ before and after different cycles of ADT. (C) CV and (D) ORR polarization curves recorded from the $Pt_{2.6}Ni/C$ before and after different cycles of ADT. The currents were normalized to the geometric area of the rotating disk electrode.

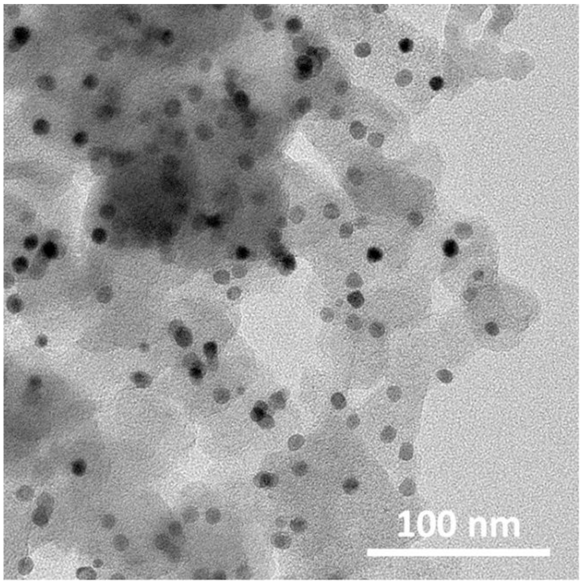

Figure S10. TEM image of the $Pt_{2.6}Ni/C$ after ADT.

Table S1. Control over the compositions (atomic percent of Ni) and shapes ($A_{\{100\}}/A_{\{111\}}$) of Pt−Ni nanocrystals, as well as conversion of $Ni(acac)_2$, by adjusting the mole fraction of $Ni(acac)_2$ added into the precursor mixture.

Mole fraction of $Ni(acac)_2$	Atomic percent of Ni (%)	Conversion of $Ni(acac)_2$ (%)	$A_{\{100\}}/A_{\{111\}}$
0.125	12.2	97.6	7.875
0.200	18.9	94.3	2.309
0.250	23.8	95.2	0.096
0.286	27. 8	97.2	0.048
0.333	31.3	93.8	0.023
0.400	38.5	96.2	0.001
0.500	45.5	90.9	0

Table S2. Electrochemically active surface area (ECSA), specific activity (SA), and mass activity (MA) of the Pt-Ni nanocrystals before and after different cycles of ADT.

Sample	Cycles of ADT	ECSA ($m^2\ g_{Pt}^{-1}$)	SA at 0.9 V ($mA\ cm^{-2}$)	MA at 0.9 V ($A\ mg_{Pt}^{-1}$)
$Pt_{1.2}Ni/C$	initial	37.4	3.13	1.17
	5000	34.1	4.19	1.43
	10000	35.5	2.76	0.98
$Pt_{1.6}Ni/C$	initial	35.1	5.33	1.87
	5000	33.2	5.84	1.94
	10000	31.8	5.19	1.65
$Pt_{2.2}Ni/C$	initial	38.3	7.15	2.74
	5000	36.7	4.17	1.53
	10000	35.2	2.70	0.95
$Pt_{2.6}Ni/C$	initial	35.8	8.66	3.1
	5000	33.5	4.21	1.41
	10000	30.9	2.65	0.82
